# Review of journal of cardiovascular magnetic resonance 2010

**DOI:** 10.1186/1532-429X-13-48

**Published:** 2011-09-13

**Authors:** Dudley J Pennell, David N Firmin, Philip J Kilner, Warren J Manning, Raad H Mohiaddin, Sanjay K Prasad

**Affiliations:** 1 CMR Unit Royal Brompton Hospital, Sydney Street, London SW3 6NP, UK; 2 National Heart and Lung Institute, Imperial College, Exhibition Road, London, SW7 2AZ, UK; 3 Department of Medicine (Cardiovascular Division) and Radiology, Beth Israel Deaconess Medical Center, 330 Brookline Avenue, Boston, MA 02215 USA; 4 Harvard Medical School, 25 Shattuck Street, Boston, MA 02115 USA

## Abstract

There were 75 articles published in the *Journal of Cardiovascular Magnetic Resonance *(JCMR) in 2010, which is a 34% increase in the number of articles since 2009. The quality of the submissions continues to increase, and the editors were delighted with the recent announcement of the JCMR Impact Factor of 4.33 which showed a 90% increase since last year. Our acceptance rate is approximately 30%, but has been falling as the number of articles being submitted has been increasing. In accordance with Open-Access publishing, the JCMR articles go on-line as they are accepted with no collating of the articles into sections or special thematic issues. Last year for the first time, the Editors summarized the papers for the readership into broad areas of interest or theme, which we felt would be useful to practitioners of cardiovascular magnetic resonance (CMR) so that you could review areas of interest from the previous year in a single article in relation to each other and other recent JCMR articles [1]. This experiment proved very popular with a very high rate of downloading, and therefore we intend to continue this review annually. The papers are presented in themes and comparison is drawn with previously published JCMR papers to identify the continuity of thought and publication in the journal. We hope that you find the open-access system increases wider reading and citation of your papers, and that you will continue to send your quality manuscripts to JCMR for publication.

## Ventricular volumes function and mass

The definition of normal values for CMR is reasonably mature, although values for special groups are still being defined,[2] but a number of research papers are still being published for assessment of less common parameters of cardiac performance as well as analysis software.

### Reference left atrial dimensions and volumes by steady state free precession cardiovascular magnetic resonance

Maceira et al published a comprehensive analysis of normal values for left atrial dimensions and volumes using steady state free precession cine imaging which is categorized by age decile, gender and body surface area[3]. This follows detailed description of normal values for the left ventricle (LV),[4] and right ventricle (RV)[5]. These data are important for clinical and research purposes as they not only provide normal values for the most commonly used cine imaging technique in current CMR use, but also they show the importance of adjusting for important covariables, which affects the categorisation into normal and abnormal. For the left atrium, the body surface area has a particularly significant effect on normal values. These values are also important for research.

### Relation between cardiac dimensions and peak oxygen uptake

Steding et al describe a study of 113 subjects of whom 71 were athletes of both genders, to test the hypothesis that total heart volume is an independent predictor of peak oxygen uptake, which is known to increase with long term endurance training and relate to left ventricular mass[6]. Multivariable analysis showed that total heart volume was a strong, independent predictor of peak oxygen uptake (R^2 ^= 0.74, p < 0.001), and as LV end-diastolic volume (EDV) increased, RVEDV increased in the same order of magnitude in both males and females (R^2 ^= 0.87, p < 0.001). The authors conclude that total heart volume is a strong, independent predictor of maximal work capacity for both males and females, and that long term endurance training is associated with a physiologically enlarged heart with a balance between the left and right ventricular dimensions in both genders.

### Peak oxygen uptake in relation to total heart volume discriminates heart failure patients from healthy volunteers and athletes

The syndrome of heart failure still raises questions, including the genesis of exercise intolerance and symptoms[7]. Engblom et al continued their examination of total heart volume as a parameter related to cardiac function by testing the hypothesis that the peak oxygen uptake to total heart volume ratio can be used to distinguish patients with heart failure from healthy volunteers and endurance athletes[8]. They studied 31 patients with various forms of heart failure, 60 healthy volunteers and 71 athletes and found that peak oxygen uptake was strongly correlated to total heart volume in the control subjects, but not for the patients. In addition, the peak oxygen uptake to total heart volume ratio differed significantly between control subjects and patients, and was the only independent predictor of presence of heart failure (p < 0.001) by multivariable analysis. The authors conclude that the peak oxygen uptake to total heart volume ratio may prove useful in early heart failure diagnosis.

### Longitudinally and circumferentially directed movements of the left ventricle studied by cardiovascular magnetic resonance phase contrast velocity mapping

Condreanu et al used high resolution CMR to detect new details of LV systolic and diastolic function, to explain the twisting and longitudinal movements of the left ventricle[9]. The authors found that left ventricular function may be a consequence of the relative orientations and moments of torque of the sub-epicardial relative to the sub-endocardial myocyte layers, with influence from tethering of the heart to adjacent structures and the directional forces associated with blood flow, and conclude that understanding the complex mechanics of the left ventricle is vital to enable these techniques to be used for the evaluation of cardiac pathology.

### Impact of diastolic dysfunction severity on global left ventricular volumetric filling - assessment by automated segmentation of routine cine cardiovascular magnetic resonance

Mendoza et al examined the relation between the severity of echocardiography derived diastolic dysfunction and volumetric filling by automated processing of routine cine CMR[10]. Automated segmentation was performed to generate diastolic filling curves from the CMR data. Comparison with echocardiography finding of diastolic dysfunction was good. The authors concluded that automated cine-CMR segmentation can discern LV filling changes that occur with increasing severity of echocardiography derived diastolic dysfunction, and that impaired relaxation is associated with prolonged filling intervals whereas restrictive filling is characterized by increased filling rates.

### Automated left ventricular diastolic function evaluation from phase-contrast cardiovascular magnetic resonance and comparison with Doppler echocardiography

Bollache et al aimed to develop a robust process to automatically estimate velocity and flow rate-related diastolic parameters from PC-CMR data and to test the consistency of these parameters against echocardiography as well as their ability to characterize left ventricular (LV) diastolic dysfunction[11]. The MR diastolic parameters varied significantly in patients with aortic stenosis as opposed to controls. Both velocity and flow rate diastolic parameters were consistent with echocardiography values (r > 0.71) and receiver operating characteristic (ROC) analysis revealed their ability to separate patients from controls, with sensitivity 80%, specificity 80% and accuracy 85%. Slight superiority in terms of correlation with echocardiography (r = 0.81) and accuracy to detect LV abnormalities (sensitivity 83%, specificity 91% and accuracy 89%) was found for the PC-CMR flow-rate related parameters. The authors conclude that their PC-CMR technique was fast and reproducible with successful extraction of consistent velocity-related diastolic parameters, as well as flow rate-related parameters. This technique provides a valuable addition to established CMR tools in the evaluation and the management of patients with diastolic dysfunction and builds on the work of Feng reported in 2009[12].

### Cardiac resynchronization therapy guided by cardiovascular magnetic resonance

Leyva has been a pioneer of the application of CMR to guide cardiac resynchronization therapy, and relate the CMR findings to hard cardiac outcomes. Previous work published in JCMR includes the visualisation of the coronary venous anatomy to guide wire placement,[13] and CMR techniques to measure dyssynchrony[14]. This authoritative state of the art review summarises his work and that of others in the field[15].

### Cardiovascular magnetic resonance in patients with pectus excavatum compared with normal controls

A number of influences on cardiac function and the electrocardiogram have been reported in JCMR, including obesity,[16] and athletic training,[17] but the influence of chest deformity is a new area. Saleh et al contribute a useful paper to the cardiology literature by studying 30 patients with pectus excavatum and determining the effects of the distorted anatomy on cardiac structure and function[18]. No significant differences between pectus excavatum patients and controls were found in LV ejection fraction, LV myocardial shortening, pulmonary-systemic circulation time or pulmonary flow indices. In pectus excavatum, resting RV ejection fraction was reduced (53.9 ± 9.6% versus 60.5 ± 9.5%; P = 0.013), RVSD was reduced (P < 0.05) both at end diastole and systole, RVLD was increased at end diastole (P < 0.05) reflecting geometric distortion of the RV due to sternal compression. The authors conclude that pectus excavatum mainly affects the right ventricle.

## Flow Evaluation and Valve Disease

The unique capability of CMR to measure cardiovascular flow is important in the armamentarium of techniques that contribute to the versatility of CMR. This has greatest application in valve disease[19,20] but it is also often used in congenital heart disease, coronary disease and pulmonary disease[21].

### Flow measurement by cardiovascular magnetic resonance: a multi-centre multi-vendor study of background phase offset errors that can compromise the accuracy of derived regurgitant or shunt flow measurements

The ability to measure the volumes of forward and regurgitant flow through planes transecting the aorta and pulmonary trunk is a unique and clinically valuable capability of CMR. However, these derived measurements of volumetric flow are sensitive to errors caused by small offsets of the measured velocities, and this has been reported previously in JCMR[22]. This multi-centre, multi-vendor study used static phantoms to test for such errors in the absence of any post-acquisition correction[23]. It found that all three types of 1.5 Tesla system tested appeared to require post-acquisition correction to achieve consistently reliable breath-hold measurements of flow. This highlighted the need for continuing work towards the minimization of such errors, a subject that has subsequently been investigated further and reported in JCMR[24].

### Baseline correction of phase-contrast images in congenital cardiovascular magnetic resonance

This paper addressed the related subject of post-acquisition phantom correction of CMR phase contrast flow velocity acquisitions on a General Electric 1.5 Tesla system, as used for aortic, pulmonary and shunt flow measurements in 149 patients in a clinical congenital CMR program[25]. Phantom correction was found, in many but not all cases, to result in clinically significant changes in flow measurements, either increased or decreased, without a consistent directionality to the changes. The study underlines the value of performing post acquisition phantom correction of flow measurements in CMR systems with known or suspected phase offset errors, as has previously been described in JCMR[26].

### Semi-automatic quantification of 4D left ventricular blood flow

The acquisition, analysis and display of multidirectional time resolved 3D or 7D flow has been well developed in JCMR[27]. Eriksson now reports the semi-automated analysis of CMR 4D flow velocity data for the quantification and visualization of flow through the LV in six normal and three dilated myopathic hearts[28]. The technique used information on cavity boundaries identified from short and long axis cine acquisitions together with the 4D flow velocity data to derive of the volumes, distributions and timings of selected components of LV flow. Such an approach may provide a basis for larger clinical research studies. It illustrates the kind of post processing software that would help 4D velocity acquisitions to become appealing for use in routine clinical investigation[29].

### Effects of gadolinium contrast agent on aortic blood flow and myocardial strain measurements by phase-contrast cardiovascular magnetic resonance

The presence or absence of gadolinium contrast agent in the blood had no demonstrable effect on phase contrast measurements of aortic flow in this study[30]. Therefore, when assessing blood flow as well as myocardial viability, time may be saved by measuring flow after gadolinium injection and before the acquisition of late gadolinium enhancement images. Phase-contrast information on myocardial displacement was also found to be measurable with or without contrast agent, but preferably undertaken pre-gadolinium as that allowed better blood-myocardial differentiation for appropriate myocardial segmentation.

### Quantification of left ventricular remodeling in response to isolated aortic or mitral regurgitation

The treatment of patients with aortic regurgitation (AR) or mitral regurgitation (MR) relies on the assessment of the severity of the regurgitation as well as its effect on LV size and function. This study set out to determine relations between regurgitant volume, measured by phase contrast velocity mapping, LV volumes and LV dimensions in patients with isolated AR or MR and preserved LV function[31]. It is unsurprising but nevertheless important that ventricular volumes were found to correlate better with regurgitant volume than linear dimensions. This paper provides valuable data on the LV volume ranges that correspond to published recommended linear dimensions for guidance on decision making regarding the timing of surgical intervention for AR or MR.

### Assessment of mitral bioprostheses using cardiovascular magnetic resonance

Direct anatomical imaging of the valves by CMR, including valve morphometry,[32] and regurgitant volumes[33,34] has become more established. This study set out to test the feasibility of CMR SSFP cine planimetry to evaluate the orifice area of mitral bioprostheses[35]. Eighteen consecutive patients, 11 in atrial fibrillation and one with frequent ventricular ectopics, were studied. The cine image appearances, the levels of agreement between CMR and transesophageal echo assessments of orifice area, and the inter-observer reproducibility of CMR planimetry measurements together provided at least preliminary support for the feasibility and potential usefulness of the CMR approach.

### Hemodynamic predictors of aortic dilatation in bicuspid aortic valve by velocity-encoded cardiovascular magnetic resonance

Bicuspid aortic valve (BAV) is a common congenital malformation which, besides its tendency to develop stenosis or regurgitation, predisposes to aortic dilatation and dissection. Compared to healthy controls, this study reports the aortic dilatation found in the group of 18 young BAV patients studied and the increased angle measured between the direction of the left ventricular outflow stream and the axis of aortic root channel[36]. These two measures were found to correlate with one another, although the causality remains unresolved. Plasma levels of matrix metallo-proteinase 2, an extracellular protein considered to be a marker of vessel wall disease, was also found to correlate with ascending aortic dilatation and the flow jet angle in the groups studied.

## Congenital and pediatric heart disease

### Assessment of atrial septal defects in adults comparing cardiovascular magnetic resonance with transoesophageal echocardiography

Pre-procedure assessment of maximal and minimal atrial septal defect (ASD) dimensions and atrial septal margins was performed by both CMR and transesophageal echocardiography (TOE) in this study[37]. CMR acquisitions included contiguous stacks of 6 mm thick SSFP cines in short and long axis orientations across the ASD region. Measurements by the two modalities agreed well with one another, so CMR was proposed as an alternative to TOE for the pre-procedure assessment of ASDs. It is worth adding that CMR can also provide reliable information on the amount of shunting and the presence or absence anomalous pulmonary veins in this common congenital condition, although these were not aspects of the study reported.

### Right ventricular ejection fraction is better reflected by transverse rather than longitudinal wall motion in pulmonary hypertension

In patients with pulmonary hypertension, fractional transverse dimensional shortening, measured from the mid septal region to the free wall of the RV in a four-chamber cine, was found to correlate better with RV ejection fraction than the fractional shortening of the long axis of the RV in this CMR study[38]. The transverse measure is therefore a potential alternative to tricuspid annular plane excursion for monitoring RV dysfunction by echocardiography, or by CMR, in pulmonary hypertension.

### Preoperative evaluation of pulmonary artery morphology and pulmonary circulation in neonates with pulmonary atresia - usefulness of MR angiography in clinical routine

Contrast-enhanced magnetic resonance angiography (CE-MRA) was found to be a useful diagnostic tool for the preoperative evaluation of the morphology of pulmonary arteries and blood supply in this retrospective study of 15 neonates with pulmonary atresia[39]. In most cases, the information provided by CMR was considered sufficient to avoid the risks of radiation exposure and the potential complications that are associated with diagnostic cardiac catheterization.

### Truncus arteriosus with aortic arch interruption: cardiovascular magnetic resonance findings in the unrepaired adult

CMR findings in an unusual case of a 28 year-old female patient with unrepaired truncus arteriosus and also interruption of the aortic arch were illustrated and described in this report[40].

## Cardiomyopathy

The use of CMR in cardiomyopathy has exploded in the last 5 years and many CMR centres now find cardiomyopathy patients form the largest proportion of the workload. The synergy of CMR with cardiovascular genetics has become clear, as has the need for CMR physicians to work closely with electrophysiology colleagues in assessing arrhythmic[41] and sudden death risks. Rare forms of cardiomyopathy,[42] which may be unclassified,[43-46] are being studied and common themes of myocardial fibrosis development, pattern of deposition, and association with outcomes are being established.

### Circumferential myocardial strain in cardiomyopathy with and without left bundle branch block

Cardiac resynchronization therapy (CRT) has been shown to improve clinical outcomes in patients with heart-failure. However, 30-40% of patients who receive CRT therapy do not show significant clinical improvement. There is much interest in refining our recognition of the latter group. In this study, Han et al sought to examine circumferential patterns in patients with LBBB and systolic dysfunction by applying tagged CMR[47]. Septal dyskinesis was as expected a frequent abnormality. Three main patterns of abnormality were seen. Some patients with LBBB had severe mechanical dyssynchrony manifested as a specific contractile pattern with initial presystolic septal contraction during isovolumic contraction period followed by dyskinesis (positive εcc) of the interventricular septum during the entire systole. This pattern was present in the anteroseptum (Type Ia) in 30% of patients, and in the entire septum in 50% of patients (Type Ib). The remaining 20% of LBBB patients had a normal contractile pattern, similar to non-LBBB cardiomyopathy patients and healthy controls, although the magnitude of contraction was significantly reduced in both groups of cardiomyopathy patients compared to healthy controls. The recognition of the presence of different mechanical contraction patterns within the same conduction abnormality may be important for the selection of patients for CRT.

### How do hypertrophic cardiomyopathy mutations affect myocardial function in carriers with normal wall thickness? Assessment with cardiovascular magnetic resonance

HCM is typically due to a sarcomeric gene mutation with an autosomal dominant pattern of inheritance. As such, early recognition of functional changes to either recognise gene carriers or as a putative target for therapy would be advantageous. In this study, Germans et al used CMR to assess global LA and LV volumes and regional intramural myocardial function in carriers with normal wall thickness[48]. The asymmetry in wall thickness between the septum and lateral wall, which is characteristic for HCM, was already present in some carriers with normal wall thickness. Typical focal LGE was present in 2 carriers. Also, LA volumes were larger in carriers. In addition, HCM mutation carriership was identified as an independent determinant of reduced circumferential strain and strain rate, which was predominantly present in the basal lateral segments. Segmental peak systolic circumferential strain (SCS) and peak diastolic circumferential strain rate (DCSR) had a high accuracy to identify carriers, but did not completely exclude HCM mutation carriership.

### Left ventricular T2 distribution in Duchenne Muscular Dystrophy

Duchenne Muscular Dystrophy (DMD) is associated with a skeletal and cardiac myopathy, the latter of which is coming under increasing scrutiny, in humans and animals,[49] as a cause of death. A better understanding of the pathobiology and early changes will provide a potential opportunity to improve management. Significant research has been done to establish a role of T2 relaxation to detect myocardial edema,[50,51] and in this study, of 26 Patients with DMD, Wansapura et al used the Full Width of Half Maximum (FWHM) of T2 distribution in the LV to quantify the myocardial structural heterogeneity in DMD patients[52]. In DMD subject groups, FWHM of the T2 histogram rose progressively with age and decreasing EF. Further, FWHM was significantly higher in those with reduced circumferential strain. The myocardial structural abnormality suggested by the observed trend is likely due to concomitant presence of interstitial fibrosis and so in the early stage is not detected by the late enhancement technique. This study supports the view that the regional dysfunction depicted by reduced circumferential strain is associated with the ultrastructural myocardial cell abnormality present in DMD patients.

### Quantitative analysis of late gadolinium enhancement in hypertrophic cardiomyopathy

The presence and amount of fibrosis in HCM appears to portend an adverse prognosis. An important practical challenge however has been defining and quantifying the presence of myocardial fibrosis by the late enhancement technique. Several methods based on a standard deviation or a full-width half maximum method have been proposed. Whilst these are highly reproducible in an infarct setting, in HCM where the fibrosis is more patchy and often diffuse, there is often the challenge of distinguishing true fibrosis from noise. In this study, Donato Aquaro and colleagues describe the merits of a cut-off derived from a Rayleigh curve as being potentially more accurate than using a fixed cut-off using a standard deviation algorithm[53]. Further work is required to compare this to other algorithms based on a full-width half maximum method.

### Right ventricular volumes and function in thalassemia major patients in the absence of myocardial iron overload

Beta-thalassemia major (TM) is a severe hereditary anemia requiring lifelong transfusions. There is a consequent iron overload, predominantly affecting the heart, liver and endocrine organs. Iron overload cardiomyopathy remains a major cause of death and therefore early detection of iron-induced cardiac toxicity is important, following by iron chelation treatment tailored to the heart[54]. In addition to measurement of myocardial T2*, ventricular function can be impaired. Much of the current work has focused on the LV, however it is well established that RV dysfunction heart-failure carries an adverse prognosis. In this study, Carpenter and colleagues extended work evaluating the LV, to define a reference range for RV volumes, ejection fraction (EF) in thalassemia major patients (TM) without myocardial iron overload[55]. This will be important in assessing the impact of myocardial iron overload in this cohort of patients and in assessing response to treatment.

### Early detection of cardiac involvement in Miyoshi myopathy: 2D strain echocardiography and late gadolinium enhancement cardiovascular magnetic resonance

The first question raised by this title must be -'so what is Miyoshi myopathy'? Miyoshi Myopathy (MM) is a distinct form of muscular dystrophy caused by mutations within the dysferlin (DYSF) gene resulting in severe to complete deficiency of dysferlin expression. Clinically, these dysferlinopathies start in young adulthood with progressive muscle weakness and atrophy that advances to severe disability in older adulthood. While the profound effect of dysferlin deficiency in skeletal muscle has been the subject of much investigation, the effect of dysferlin deficiency in cardiac muscle have not been studied yet. In this study, Choi et al, demonstrated a reduction in longitudinal strain and the presence of replacement fibrosis in a subset of affected patients[56]. This was detected prior to the development of cardiovascular symptoms or a reduction in overall LVEF. The ramifications are two-fold. Firstly in this uncommon myopathy demonstrating an opportunity for early detection. Secondly and more broadly, it extends the 'portfolio' of cardiomyopathies where CMR in conjunction with echo provides unique insights based on tissue characterization.

### Troponin release following endurance exercise: is inflammation the cause? A cardiovascular magnetic resonance study

It is generally acknowledged that exercise is a good thing. Yet debate persists about the true health benefits of ultra-endurance forms exercise - particularly marathon running where a troponin rise is seen - often to the same levels as an acute myocardial infarction. In this study, O'Hanlon and colleagues examined if there were detectable tissue changes on CMR in a cohort of volunteers scanned following a marathon[57]. A baseline control scan pre-exercise was performed in all participants. Exercise induced cardiac biomarker release was not associated with any functional changes by CMR or any detectable myocardial inflammation or fibrosis. This study contributes to understanding the link, aetiology and significance of the troponin rise post-exercise.

### Epicardial adipose tissue in patients with heart failure

The role of epicardial adipose tissue (EAT) and its contribution to the development of cardiac pathology is quite ambiguous. There is growing evidence of a close functional and anatomical relationship between the adipose tissue and muscular components of the heart. Its close proximity to the myocardium suggests that EAT is a metabolically active organ and a source of several bioactive molecules may influence cardiac morphology and function. In this study, Doesch et al demonstrate that in patients with CHF and severely reduced impaired LV-EF (LV-EF < 35%), EAT is significantly reduced compared to healthy controls[58]. The reduction of EAT is irrespective of the underlying aetiology of the cardiomyopathy. Like in healthy controls an increase in left ventricular mass also leads to an augmentation of the EAT mass in patients with CHF, however, contrary to previous studies, the EAT mass/LV-EDM ratio is significantly lower compared to healthy controls. Abnormalities and/or anatomic alterations due to disturbed cardiac function and geometry seem to play a key role and are a possible explanation for these findings.

### Myocardial late gadolinium enhancement cardiovascular magnetic resonance in patients with cirrhosis

End stage liver disease (ELD) is associated with major alterations in the regulation of the cardiovascular system. Portal hypertension and/or hormonal changes in ELD induce a hyperdynamic circulatory state characterized by arterial hypotension and tachycardia and are often accompanied by ascites and electrolyte disturbances. Recent data also emphasizes the impact of liver function on renal (hepatorenal syndrome) and pulmonary (hepatopulmonary and portopulmonary syndrome) circulation. Whilst there is much focus on the causes and treatment of renal and pulmonary manifestations, relatively little is understood about the myocardial changes as a result of ELD. In this study, Lossnitzer et al show that myocardial alterations have a high prevalence among ELD patients[59]. This is often in a pattern seen in myocarditis. Commonly identified features were a hyperdynamic LV function and a patchy pattern of replacement fibrosis. This work is useful in furthering our understanding of the effects of severe liver disease and may have potential value in guiding selection of Patients for liver transplantation - the latter needs larger prospective trials.

### Accuracy of cardiovascular magnetic resonance in myocarditis: comparison of MR and histological findings in an animal model

In the evaluation of myocarditis, CMR has now frequently replaced the more traditional method of endomyocardial biopsies (EMB) to confirm the diagnosis. As well as being invasive and with a tangible risk of severe morbidity or mortality, EMB has frequently suffered from sampling bias resulting in a low sensitivity. In an animal model, the extent of LGE correlated to the histological severity of myocarditis and to serum-levels of troponin T on day 21[60]. Areas of LGE had nearly identical topographic distribution as compared to histologically proven areas of inflammation and provided a good marker of cardiomyocyte necrosis. This is supportive data for the clinical use of CMR either as an alternative to EMB, or where it is important to know the pathogen, to guide the site of EMB.

### Myocardial fibrosis in desmin-related hypertrophic cardiomyopathy

Desmin is the main intermediate filament protein expressed in skeletal, cardiac, and smooth muscle. It interacts with other proteins to form a continuous cytoskeletal network that maintains a spatial relationship between the contractile apparatus and other structural elements of the cell, thus providing maintenance of cellular integrity, force transmission, and mechano-chemical signalling. Primary desminopathies are caused by mutations in the desmin gene. This disease is characterized by an intracellular accumulation of insoluble protein aggregates eventually leading to cell death and replacement fibrosis. In this case report, fibrosis was detected by LGE in the absence of global or focal systolic wall motion abnormalities[61]. LGE may have value therefore in the diagnosis and early evaluation of affected individuals.

## Atheroma and Vascular

Many experts consider that CMR will play an increasing role in characterisation of the atherosclerotic arterial wall,[62] with a focus on early detection, monitoring of response to treatment,[63] and relation to outcomes,[64] rather than a slavish attention of stenosis detection for which other techniques are widely used. Vessel wall CMR,[65,66] and angiography,[67,68] seem to clearly benefit from the use of 3 T. The papers in this section illustrate the variety of ways that CMR can be used to investigate vascular disease.

### Variations in atherosclerosis and remodelling patterns in aorta and carotids

This is an interesting study that attempts to examine atherosclerosis progression and regression in multiple vascular beds (thoracic aorta, abdominal aorta and carotids) of 28 patients using black blood CMR over a one year period[69]. Luminal and wall areas were measured. Results of this study indicate that different vascular locations exhibited varying progression of atherosclerosis and remodelling as monitored by CMR. However there are a number of important limitations to be noted including the retrospective nature of the analysis on a small number of patients, and plaque burden was measured across the entire artery and not within specific plaque. Other limitations are linear model and not multivariate regression analysis was used.

### Thoracic aortopathy in Turner syndrome and the influence of bicuspid aortic valves and blood pressure: a CMR study

This is an observational, cross-sectional study of aortic dimensions within a large, unselected cohort of adult patients with Turner's syndrome (TS)[70]. As such, this study gives important and valuable "normal" data for this specific cohort, and describes prominent risk factors for abnormal aortic dimension. A higher incidence of aortic valve disease, aortopathy, aortic coarctation and dissection has been well-described in multiple prior population studies. The current manuscript, however, attempts to more rigorously describe the size characteristics of a "general population" of TS patients in comparison to a control group - utilizing both CMR techniques and echocardiography. Overall, the study provides important information in a systematic fashion, and adds to the literature of TS central aortic disease.

### Ultra-short echo time cardiovascular magnetic resonance of atherosclerotic carotid plaque

This small ex vivo study examines the feasibility of detecting carotid plaque calcification using an ultra-short TE (UTE) technique[71]. Fourteen ex-vivo human plaques with UTE MR, CT and histology and found a reasonable agreement among the three approaches for calcification identification. The authors also noticed a certain amount of false positive readings from UTE MR when compared to CT and histology. Although the authors identified a certain degree of agreement between UTE and histology, no definite conclusion could be drawn without supporting quantitative comparisons. Another concern is the possible mismatch between MR, CT and histology.

### Measuring aortic pulse wave velocity using high-field cardiovascular magnetic resonance: comparison of techniques

The authors quantitatively compare three different approaches for aortic pulse wave velocity measurements at 3 T including transit time, flow area measurements and the cross correlation method[72]. Fifty heterogenous patients and 6 healthy volunteers were scanned and the inter-observer, intra-observer and the study-restudy variability were reported. Each method has advantages and limitations but no bias among the three methods was found although the flow area method was found to be the least reproducible.

### Natural history of spontaneous aortic intramural hematoma progression: Six years follow-up with cardiovascular magnetic resonance

This is an interesting case report on the natural history and evolution of spontaneous intramural haematoma (IMH) in a patient with good quality images and literature review[73]. The IMH followed for 6 years by CMR imaging. The patient progressed through different stages, including hematoma absorption, ulcer-like lesion emergence, aneurysm enlargement and limited aortic dissection.

### The association of lesion eccentricity with plaque morphology and components in the superficial femoral artery: a high-spatial-resolution, multi-contrast weighted CMR study

The authors describe the association between atheromatous plaque eccentricity with morphology and composition in the superficial artery assessed by multicontrast weighted-CMR[74]. The study involved 28 subjects and 180 diseased segments. All patients had an ankle-brachial index < 1.00. The authors concluded that eccentric lesions were larger despite having similar luminal area. In addition, they contained larger lipid rich necrotic core and more calcification. The study is of interest but is limited by being observational with low number of patients and significant variability in PAD severity.

### Regional in vivo transit time measurements of aortic pulse wave velocity in mice with high-field CMR at 17.6 Tesla

This study describing a phantom and mouse set of experiments validating the transit time method by MR phase velocity encoding for measuring pulse wave velocity using 17.6 T scanner[75]. The authors show validation of the method in the phantom and that PWV is higher in APO-E KO mice than controls. Transgenic mouse models are increasingly used to study the pathophysiology of human cardiovascular diseases. The aortic pulse wave velocity (PWV) is an indirect measure for vascular stiffness and a marker for cardiovascular risk.

### Right coronary wall CMR in the older asymptomatic advance cohort: positive remodelling and associations with type 2 diabetes and coronary calcium

CMR Black blood coronary wall imaging was used in 223 elderly patients without known history of cardiovascular disease to evaluate coronary wall CMR in an asymptomatic older cohort[76]. Image quality was fair or good in 67% of patients allowing for assessment of vessel, wall and lumen area as well as vessel wall thickness in 150 subjects. Multivariate analysis showed association between total/HDL cholesterol ratio and wall thickness as well as between diabetes and vessel area and wall thickness. Furthermore, the authors report significant correlation between calcium score, vessel area, wall area, and wall thickness. Bland-Altman analysis demonstrated only small differences for inter and intra-observer measurements of vessel area, wall area, and lumen area. The authors conclude that coronary wall CMR may contribute to the non-invasive assessment of subclinical coronary atherosclerosis in older, at-risk patient groups.

## Perfusion

Perfusion CMR continues to grow, and new steps in optimisation have been published including accelerated acquisition, high field CMR, and improved analysis including quantification. In many centres, perfusion CMR is making inroads into established referral patterns for nuclear based techniques. Progress in perfusion CMR in children and women in particular has occurred in a desire to lower radiation burden in these sensitive individuals[77]. However there remains room for improvements in ease of analysis and quantification, artefact elimination,[78] robustness and relation to outcomes[79].

### Evaluation of contrast wash-in and peak enhancement in adenosine first pass perfusion CMR in patients post bypass surgery

Kelle et al performed perfusion CMR on 38 patients after coronary bypass grafting (CBG) without coronary obstruction and compared the results with 20 patients with no obstructive coronary disease at coronary angiography to determine whether differences in epicardial wash-in kinetics were present[80]. In areas perfused by coronary arteries with bypasses compared to native coronaries, the time for contrast to reach maximum in native coronaries and bypasses was 12.6 s ± 3.0 s vs 13.1 s ± 3.0 s (p < 0.05), respectively. The delay in Tmax resulted in a significant (p < 0.05) delay of 0.5 ± 1.1 heart beats (= images) when adjusted to the heart rate. Differences in time were most pronounced in areas perfused by left internal mammary artery grafts rather than by venous CBG, but were also present between native vessel territories in patients without CAD, albeit with smaller variability. The authors conclude that adenosine perfusion CMR in patients post CBG may be associated with a short delay in contrast arrival, but that this does not seem to be a limiting factor for the accuracy of first pass adenosine perfusion in patients post CBG.

### Meta-analysis of the diagnostic performance of stress perfusion cardiovascular magnetic resonance for detection of coronary artery disease

Hamon et al performed a meta-analysis of the performance of perfusion CMR summarising 35 original articles fitting pre-specified inclusion criteria including 1.5 T imaging and comparison with coronary angiography[81]. From the 263 citations identified, 55 relevant original articles were selected. The overall patient-based analysis demonstrated a sensitivity of 89% (95% CI: 88-91%), and a specificity of 80% (95% CI: 78-83%). Adenosine stress perfusion CMR had better sensitivity than with dipyridamole (90% (88-92%) versus 86% (80-90%), P = 0.022), and a tendency to a better specificity (81% (78-84%) versus 77% (71-82%), P = 0.065). The authors conclude that perfusion CMR is highly sensitive for detection of CAD but its specificity remains moderate.

### Real-time cine and myocardial perfusion with treadmill exercise stress cardiovascular magnetic resonance in patients referred for stress SPECT

Raman et al performed an unusual and interesting perfusion CMR study, using treadmill stress and real-time CMR in a cohort of 43 patients referred for perfusion SPECT[82]. Using an ingenious protocol of technetium injection at peak exercise and CMR immediately post-exercise they were able to complete the real-time CMR in 88 seconds with only a 68 second delay after exercise. Agreement between SPECT and CMR was moderate (κ = 0.58). Accuracy in eight patients who underwent coronary angiography was 7/8 for CMR and 5/8 for SPECT (p = 0.63). Follow-up at 6 months indicated freedom from cardiovascular events in 29/29 CMR negative and 33/34 SPECT-negative patients. The authors conclude that exercise stress CMR including wall motion and perfusion is feasible in patients with suspected ischemic heart disease and that further trials may be warranted.

### Reproducibility of adenosine stress cardiovascular magnetic resonance in multi-vessel symptomatic coronary artery disease

Chih et al performed an important study examining the interstudy reproducibility (test-retest repeatability) of adenosine stress perfusion CMR in 20 patients (10 with coronary disease and 10 at low risk for coronary disease)[83]. The CoV for the number of ischemic segments was 31% with a mean difference of -0.15 ± 0.88 segments and 91% perfect agreement between studies. The reproducibility of MPRi was 19% with no significant difference between patients with CAD and those with low risk CAD (p = 0.850). For trials using perfusion CMR as an endpoint, an estimated sample size of 12 subjects would be required to detect a two-segment change in the number of ischemic segments (power 90%, α 0.05). The authors conclude that adenosine stress CMR, by qualitative and semi-quantitative normalized upslope analyses are reproducible techniques in both patients with multi-vessel CAD and those without known CAD. The robust inter-study reproducibility of perfusion CMR supports its clinical and research application.

### Quantification of myocardial perfusion using CMR with a radial data acquisition: comparison with a dual-bolus method

Kim et al explored the use of radial data acquisition at 3 T for quantification of perfusion CMR[84]. Using a dual bolus approach, the arterial input function (AIF) was calculated from the blood signal in three sub-images with differing effective saturation recovery times (SRT). The full and sub-images were reconstructed iteratively with a total variation constraint. The images from the full 72 ray data were processed to obtain tissue enhancement curves. A 2-compartment model was used to determine absolute flows. The proposed multi-SRT method resulted in AIFs that were similar to those obtained with the dual-bolus method. The authors conclude that the multi-SRT method with a radial k-space perfusion sequence, can be used to obtain an accurate AIF and thus quantify myocardial perfusion for doses of contrast agent that result in a relatively saturated AIF.

### Quantification of myocardial perfusion by cardiovascular magnetic resonance

Jerosch-Herold has pioneered many aspects of perfusion CMR, and in this authoritative review he summarises the quantification of myocardial perfusion using first pass techniques[85]. The potential of contrast-enhanced cardiovascular magnetic resonance (CMR) for a quantitative assessment of myocardial perfusion has been explored for more than a decade, with encouraging results from comparisons with accepted "gold standards", such as microspheres used in the physiology laboratory. This has generated an increasing interest in the requirements and methodological approaches for the non-invasive quantification of myocardial blood flow by CMR. The field has reached a stage, where quantification of myocardial perfusion is no longer a claim exclusive to nuclear imaging techniques. CMR may in fact offer important advantages like the absence of ionizing radiation, high spatial resolution, and an unmatched versatility to combine the interrogation of the perfusion status with a comprehensive tissue characterization. Further progress will depend on successful dissemination of the techniques for perfusion quantification among the CMR community.

### Nasal continuous positive airway pressure improves myocardial perfusion reserve and endothelial-dependent vasodilation in patients with obstructive sleep apnea

Nguyen et al studied whether obstructive sleep apnoea (OSA) as a risk factor for coronary artery disease, by measuring myocardial perfusion and brachial artery reactivity in a randomised controlled study of 35 OSA patients randomised to 3 months of nasal continuous positive airway pressure (nCPAP) or sham nCPAP[86]. Patients on nCPAP showed improved perfusion and vascular reactivity compared to those on sham treatment. The authors conclude that that relief of apnea in OSA may improve microvascular disease and endothelial dysfunction, which may prevent the development of overt cardiovascular disease, and that further study in a larger patient population, may be warranted.

### Feasibility and safety of high-dose adenosine perfusion cardiovascular magnetic resonance

Karamitsos et al studied the use of high-dose adenosine for perfusion CMR in patients who showed no haemodynamic response to adenosine at the normal dose of 140 ug/kg/minute[87]. In a study of 98 patients, 18 satisfied the entry criteria for response failure and the infusion dose was increased to 210 ug/kg/min at which point 16 of the 18 patients showed a response. The authors concluded that increased age or reduced ejection fraction were predictors of non-response, and presumably undisclosed drinking of caffeine was also a possibility. The study was not large enough to examine effects on diagnostic accuracy but a further trial might address this issue.

## Acute Coronary Syndrome

Increased research in acute coronary syndromes has been driven by the ability of CMR to look at a number of phenomena that are difficult or impossible to image by other in-vivo techniques. This includes microvascular obstruction, myocardial edema, myocardial salvage and relation of findings to wall motion[88]. There is optimism that this will translate into clinical trials of adjuvant therapies to standard primary percutaneous coronary intervention.

### Staged cardiovascular magnetic resonance for differential diagnosis of Troponin T positive patients with low likelihood for acute coronary syndrome

Troponin biomarkers are very sensitive of myocardial infarction but patients sometimes present with an elevated troponin in the setting of inconclusive symptoms and ECG-changes. Confirmation of acute coronary syndrome (ACS) or accurate non-ACS diagnosis is of paramount importance to avoid unnecessary invasive procedures and to guide therapy. Steen et al reported on the role of CMR in 29 such patients with elevated troponin yet low-intermediate probability of IHD[89]. A comprehensive successive eight-step CMR (cine, perfusion, T2 weighted (T2w), pulmonary angiography, and late gadolinium enhancement (LGE) was performed at 1.5 T and characterized 93% of the elevated troponin cases - including 38% with ACS, 21% with pulmonary embolism, 17% with myocarditis.

### Cardiovascular magnetic resonance of the myocardium at risk in acute reperfused myocardial infarction: comparison of T2-weighted imaging versus the circumferential endocardial extent of late gadolinium enhancement with transmural projection

The use of CMR for identification of myocardium at risk (MaR), the myocardium supplied by the occluded vessel that is subject to ischemia has received considerable recent attention. Ubachs et al compared short-axis triple inversion turbo spin echo T2w imaging with LGE for assessment of MaR in 37 patients with early reperfused first-time ST segment elevation myocardial infarction (STEMI) within a week of percutaneous coronary intervention[90]. T2w MaR was nearly 50% greater than MaR derived from LGE, with only a modest correlation of the methods. As a result, myocardial salvage derived from T2w imaging was similarly greater.

### Assessment of myocardium at risk with contrast enhanced steady-state free precession cine cardiovascular magnetic resonance compared to single-photon emission computed tomography

In another ACS study, Sorensson et al studied MaR derived from the contrast enhanced cine steady-state-free precession (SSFP) CMR compared with single photon emission computer tomography (SPECT) as the gold standard[91]. Sixteen patients with STEMI due to a total coronary occlusion were studied. Prior to opening the occluded vessel, patients received 99mTc tetrofosmin with imaging performed within 4 hours. CMR was performed within a week with gadolinium-DTPA administered before acquisition of short-axis SSFP cines. Contrast enhanced myocardium in SSFP cines was manually segmented. There was a very good correlation (r2 = 0.78) with very small (0.5 +/- 5.1%) difference between methods. An obvious advantage of the post-Gd cine SSFP method is that it does not require a second focused sequence.

### Relationship of dysglycemia to acute myocardial infarct size and cardiovascular outcome as determined by cardiovascular magnetic resonance

Mather et al applied LGE CMR to examine the impact of dysglycemia on acute myocardial infarction (AMI) size in 93 patients presenting with their first AMI[92]. Patients with dysglycermia (admission blood glucose ≥ 7.8 mmol/l but < 11.1 mmol/l) as well as those with diabetes mellitus (prior history or admission glucose ≥ 11.1 mmol/l) were more likely to have near (> 75%) transmural infarcts, both during the index admission and at a median follow-up of 11 months. Early LGE evidence of microvascular obstruction and left ventricular ejection fraction (LVEF) were similar.

### Differentiation of acute and four-week old myocardial infarct with Gd(ABE-DTTA)-enhanced CMR

Kirschner et al used a novel CMR contrast agent gadolinium, Gd (ABE-DTTA), to differentiate acute vs. chronic (one month old) myocardial infarction in a dual infarction canine model[93]. Gd ABE-DTTA) led to enhancement of only the acute infarction, whereas conventional Gd-DTPA was associated with enhancement of both acute and chronic infarcts.

## Chronic Ischemic Heart Disease

Since the seminal clinical publication by Kim et al over a decade ago,[94] LGE CMR has played an increasing role in the management of chronic IHD. The use of LGE has transformed the investigation and clinical practice of chronic coronary disease, and yielded considerable new insights into infarction[95,96]. Work is still progressing on how best to quantify LGE in relation to outcome,[97] and the relative merits versus dobutamine stress CMR[98]. The JCMR papers presented examine important aspects of this field.

### Prediction of global left ventricular functional recovery in patients with heart failure undergoing surgical revascularisation, based on late gadolinium enhancement Cardiovascular Magnetic Resonance

Pegg et al extended this work by examining whether viable or the sum of viable plus normal segments best predicted recovery of global LVEF in 33 patients undergoing coronary artery bypass grafting (CABG)[99]. Overall, LVEF improved from 38 to 47%, but the only independent predictor for and LVEF improvement of ≥ 3% was the number of viable *plus *normal segments. Receiver operator characteristic analysis demonstrated that at least 10 viable plus normal segments best predicted an LVEF improvement of ≥ 3%.

### Relation between regional and global systolic function in patients with ischemic cardiomyopathy after β-Blocker therapy or revascularization

Novel insights into the mechanism of ventricular remodelling to betablocker and mechanical revascularization were reported by Kaandorp et al who studied 32 patients with chronic IHD before and 8 months after assignment to betablocker or revascularization therapy[100]. In both groups, resting LVEF improved and LV end-systolic volume declined. However, stepwise multivariate analysis demonstrated that LVEF improvement in the betablocker group was related to improved function of remote myocardium, whereas in the revascularized group, improved function was noted in the dysfunctional and adjacent regions.

### Improvement of myocardial perfusion reserve detected by cardiovascular magnetic resonance after direct endomyocardial implantation of autologous bone marrow cells in patients with severe coronary artery disease

CMR has been used to monitor the success of stem cell treatment in animals,[101] but human use in clinical trials has been sparse with mixed results. Chan et al used CMR methods to examine LVEF and myocardial perfusion reserve in 12 patients with chronic IHD after autologous bone marrow cell implantation[102]. An average of 16 injections per patient were performed. Patients randomized to receive bone marrow cell injection had a significant decrease in the peri-infarct region and an increase in regional wall thickening, global LVEF, and myocardial perfusion reserve over the target area at 6 months.

### The 20 year evolution of dobutamine stress cardiovascular magnetic resonance

A highlight of the 2010 JCMR publications was an comprehensive review of dobutamine stress CMR by Charoenpanichkit and Hundley, a pioneer and ongoing leader in the field[103]. The review provides comprehensive information for the novice and expert stress CMR practitioner regarding pharmacology, safety, protocols/methods, clinical applications, accuracy, and outcome data.

## Electrophysiology and Interventional

### Extent of late gadolinium enhancement detected by cardiovascular magnetic resonance correlates with the inducibility of ventricular tachyarrhythmia in hypertrophic cardiomyopathy

Fluechter et al investigated the role of myocardial fibrosis detected by late gadolinium-enhancement (LGE) CMR as a potential arrhythmogenic substrate in 76 consecutive patients with HCM[104]. Of these patients, 43 had 1 or more risk factors for sudden cardiac death and were therefore clinically classified as high-risk patients. Of these 43 patients, 38 additionally underwent an electrophysiological (EP) testing. The high-risk patients had a significant higher prevalence of LGE than low-risk patients (67% vs 47%; p = 0.03). Also the % of LV mass with LGE was significantly higher in high-risk patients than in low-risk patients (14% vs 3%, p = 0.001). Of the 38 high-risk patients, 12 had inducible VT and these patients had significantly higher % LGE (22% vs 10%, p = 0.03, but LGE prevalence was comparable between HCM patients with and those without inducible VT (83% vs 58%; p = 0.12). In the univariate analysis the % of LV mass with LGE and the septal wall thickness were significantly associated with the high-risk group (p = 0.001 and 0.004, respectively). Multivariate analysis demonstrated that the extent of LGE was the only independent predictor of the risk group (p = 0.03). The authors conclude that the extent of fibrosis may serve as potential arrhythmogenic substrate for the occurrence of VT, especially in patients with clinical risk factors for SCD

### Towards real-time cardiovascular magnetic resonance-guided transarterial aortic valve implantation: In vitro evaluation and modification of existing devices

Cardiovascular magnetic resonance (CMR) is attractive for real-time device placement and intervention,[105] but solutions for heating and metallic artefacts are needed[106]. CMR might be an attractive alternative for guiding transarterial aortic valve implantation (TAVI) featuring unlimited scan plane orientation and unsurpassed soft-tissue contrast with simultaneous device visualization. Kahlert et al sought to evaluate the CMR characteristics of both currently commercially available transcatheter heart valves (Edwards SAPIEN™, Medtronic CoreValve^®^) and a custom-built, CMR-compatible delivery device for the Medtronic CoreValve^® ^prosthesis as an initial step towards real-time CMR-guided TAVI[107]. The devices were systematically examined in phantom models on a 1.5 T scanner using high resolution T1-weighted 3D FLASH, real-time TrueFISP and flow-sensitive phase-contrast sequences. Major susceptibility artifacts were present for the 2 commercial delivery devices precluding in-vivo application. By contrast, the nitinol-based Medtronic CoreValve^® ^prosthesis was well visualized with good visualization during catheter movement and valve deployment on real-time TrueFISP imaging. Reliable flow measurements could be performed for both stent-valves after deployment using phase-contrast sequences. The authors conclude that the novel prosthesis is potentially suited for real-time CMR-guided placement in vivo after suggested design modifications of the delivery system.

## Technical advances and new techniques

The editors of JCMR continue to support publication of new CMR techniques and the recent review on diffusion spectral imaging (DSI) with application to tractography of *ex vivo *animal hearts proved very popular[108]. The new techniques described in this section are of interest especially to the CMR physics community for translation into robust new human tools.

### Deformation analysis of 3D tagged cardiac images using an optical flow method

Myocardial tagging still presents technical challenges in implementation and analysis[109,110]. This study describes a method for measuring strain using MR imaging with tags in three dimensions coupled to analyses based on optical flow[111]. The initial part of the study involved imaging an ex vivo sheep's heart, the images of which were processed to introduce tags and known motion. These images with their known motion were then used to validate the optical flow motion measurement. These motion simulations were used to optimise the required tag space and angles for the later in vivo validation. Optical flow motion estimates obtained *in vivo *by 3D tagging during systole were validated by comparison with more conventional analysis and measurements of myocardial strain.

### Analytical method to measure three-dimensional strain patterns in the left ventricle from single slice displacement data

The paper describes a means of estimating the full 3D strain tensor from a slice displacement encoded in three orthogonal directions, and including assumptions about the through-plane shear components and compressibility of the tissue[112]. The analytical method estimates the out-of-plane as well as the in-plane components of the Lagrangian strain tensors of the myocardium from time series of MR images containing only one slice. The goal of this technique is to reduce the amount of data acquisition when calculating the strains from cardiac image. Various tests of the method are performed for the validation of the method, both on simulated and real data. The behaviour of the technique with regard with the noise level is presented. The feasibility of the method is demonstrated in a healthy human subject and the results are compared to those of other studies.

### Real-time cardiovascular magnetic resonance at high temporal resolution: radial FLASH with nonlinear inverse reconstruction

This paper demonstrates initial results of an approach providing real-time CMR, the data from which can be retrospectively reconstructed to provide high resolution[113]. The method uses undersampled radial FLASH at 3 T with non-linear inversion reconstruction. Image acquisition times were as short as 20 to 30 ms. With potential future increases in processing speed this method promises to enable spectacularly high spatial temporal resolution imaging for a range of cardiovascular applications.

### Accelerated cardiovascular magnetic resonance of the mouse heart using self-gated parallel imaging strategies does not compromise accuracy of structural and functional measures

The huge increase in the development of transgenic mouse models has made mouse phenotyping an increasingly important topic, and basic issues such as sedation and anaesthesia can affect these tiny hearts[114]. In this manuscript, self-gating and parallel imaging (SENSE) techniques were combined to reduce scan time in retrospectively gated mouse heart CMR at 4.7 T[115]. Although well-established in humans, parallel imaging in rodents is much less developed because of their very high heart-rates, intrinsic low SNR and the limited availability of phased-array coils. Both healthy and infarcted mice were subjected to cine-MRI using different acceleration factors (i.e. 1-3). Left ventricular volumes and functional parameters obtained from accelerated data sets were compared to fully sampled reference data. Results revealed only minor differences in image quality of short- and long-axis cardiac cines: small anatomical structures were accurately detected even for 3-fold accelerated data acquisition using a four-element phased array coil. The authors concluded that the accuracy of structural and functional parameters of the mouse heart was not compromised by the application of the described accelerated data acquisition method.

### An isolated perfused pig heart model for the development, validation and translation of novel cardiovascular magnetic resonance techniques

This article by Schuster et al is of significant interest to the CMR community[116]. While perfused heart models have been widely used for metabolic studies, using MRI spectroscopy, the same is not true for imaging applications. A newly developed isolated pig heart model will open important new avenues of validating novel CMR sequences (eg. absolute myocardial blood flow quantification) and will allow testing of novel treatment strategies (e.g. ischaemia reperfusion injury interventions). The authors describe this heart model to be stable and illustrate it's use on both 1.5 and 3 T clinical scanners. Numbers were small in this pilot study but there appears to be a very good agreement between CMR measures of blood flow and late gadolinium enhancement following selective alterations of coronary blood flow, which fits with the absence of coronary collaterals in the pig heart.

### BOLD cardiovascular magnetic resonance at 3.0 Tesla in myocardial ischemia

This paper describes a novel approach to detect stress-induced myocardial ischemic reaction using CMR T2* at 3 T in patients with suspected or known coronary artery disease (CAD)[117]. With quantitative coronary angiography as the reference standard, this study demonstrated the feasibility of myocardial T2* measurement at 3 T to differentiate between ischemic, non-ischemic, and normal myocardial segments in a small patient population. In conclusion, the authors stated that Stress T2* at 3 T was capable of identifying patients with significant CAD.

### Cardiovascular magnetic resonance at 3.0 T: Current state of the art

This manuscript represents a useful review of the application of 3 T CMR and how this differs from 1.5 T[118]. It is well written and complete particularly with regard to the clinical applications, adopts a top-level approach to the physics and overall provides a balanced perspective. As might be expected there is little in the way of conclusions except that 3 T is good for some things, but typically has more artefacts.

### Elasticity-based determination of isovolumetric phases in the human heart

In this paper, the authors present an investigational study on a potential application of MR-Elastography (MRE) in cardiology,[119] and the data continues to develop previously published work in this area in JCMR[120]. The method attempts to quantify time delays associated with isovolumetric tension and relaxation based on delays between volume-time and shear wave amplitude-time curves. The isovolumetric tension time was shown to be statistically significantly longer in those patients with known myocardial relaxation abnormalities. The hope is that this time will prove to be correlated to disease stage and type and therefore this could have a significant impact both in terms of diagnosis but also monitoring of patients with those cardiac diseases which are known to induce changes in myocardial stiffness.

### Acoustic cardiac triggering: a practical solution for synchronization and gating of cardiovascular magnetic resonance at 7 Tesla

This paper describes the use of the acoustic sensing approach to gating CMR data acquisitions[121]. The method is applied at 7Tesla where ECG gating can be challenging, and compares this new acoustic approach with the well proven vector ECG and Pulse-oxymetry methods. Standard parameters including end-systolic volume (ESV), end-diastolic volume (EDV), ventricular mass and ejection fraction as well as subjective scores of image quality are compared for the three triggering methods in healthy subjects. Results indicate that the acoustic method is an improvement over ECG and offers some advantages of Pulse-oxymetry and that it provides a feasible approach to cardiac gating at 7 T.

### Shortened Modified Look-Locker Inversion recovery (ShMOLLI) for clinical myocardial T1-mapping at 1.5 and 3 T within a 9 heartbeat breathhold

This manuscript is of interest to the CMR community, since many advanced CMR sites are currently moving to quantitative techniques for the assessment of myocardial injury[122]. The authors present a modified version of the MOLLI T1 mapping technique. The proposed method could offer help for some patients with breath-hold difficulty as it requires shorter acquisition times than the original technique (9 vs. 17 heart beats). The approaches are compared in simulations, phantoms, and 10 healthy volunteers at 1.5 T and 3 T. Finally, feasibility is also demonstrated in 4 patients with acute myocardial infarction.

### Cardiovascular magnetic resonance physics for clinicians: part I

This is an excellent introduction into the basic physics relating to cardiovascular magnetic resonance[123]. It reviews the MR principles underlying T1 and T2 relaxation and net magnetization, describes the components of the scanner (Main field, gradients, rf coils), describes image acquisition (slice select, frequency encoding, phase encoding), pulse sequences (GRE, SE, TSE), as well as issues related to cardiac implementation. The text is very well written and is easy to read and follow due to the methodical way in which it is explained. Even the more difficult concepts described later in the paper are described very well and are easy to follow. Unlike many physics texts that can be dominated by too much basic physics that are not relevant to a basic understanding of how the system works, even the more difficult concepts are explained with reasoning behind their use and practical examples which help the explanation.

## Varia

For busy practitioners of CMR, there is a well recognised source of referrals which is simply put as unusual pathology, or cases where other imaging has failed to yield a definitive diagnosis, or where a research technique might have clinical application. These include pericardial disease,[124,125] tumours,[126] or inflammatory diseases,[127] amongst others. We therefore include this section on papers and also include official reports, guidelines and editorials,[128-131] which are not readily categorized.

### Oxygenation-sensitive CMR for assessing vasodilator-induced changes of myocardial oxygenation

As myocardial oxygenation may serve as a marker for ischemia and microvascular dysfunction, it could be clinically useful to have a non-invasive measure of changes in myocardial oxygenation, however the impact of induced blood flow changes on oxygenation is not well understood. Vohringer et al used oxygenation-sensitive CMR to assess the relation between myocardial oxygenation, coronary sinus blood oxygen saturation (SvO2) and coronary blood flow in a dog model in which hyperemia was induced by intracoronary administration of vasodilators[132]. During acetylcholine and adenosine injection, CMR signal intensity correlated linearly with simultaneously measured SvO2 (r2 = 0.74, P < 0.001). Both SvO2 and CMR signal intensity were exponentially related to coronary blood flow, with SvO2 approaching 87%. The authors concluded that oxygenation-sensitive CMR may be useful to assess ischemia and microvascular function in patients and that its clinical utility should be evaluated.

### Quantification of global myocardial oxygenation in humans: initial experience

McCommis et al assessed the feasibility of new CMR methods to quantify global and/or regional myocardial oxygen consumption rate (MVO2) at rest and during pharmacologically induced vasodilation in 6 normal volunteers[133]. A breath-hold T2 quantification method was developed to calculate) and MVO2 rate at rest and/or during hyperemia, using a two-compartment model. A previously reported T2 quantification method using turbo-spin-echo sequence was also applied for comparison. The T2 quantification method yielded a hypaeremic OEF of 0.37 ± 0.05 and a hyperaemic MVO2 of 9.2 ± 2.4 μmol/g/min. The corresponding resting values were 0.73 ± 0.05 and 5.2 ± 1.7 μmol/g/min respectively, which agreed well with published literature values. The MVO2 rose proportionally with rate-pressure product from the rest condition. The T2 sensitivity was approximately 95% higher with the new T2 method than turbo-spin-echo method. The authors conclude that the CMR has potential for non-invasive estimation of myocardial oxygenation.

### Quantitative cardiovascular magnetic resonance for molecular imaging

Winter et al review the growing field of CMR molecular imaging, which aims to identify and map the expression of important biomarkers on a cellular scale utilizing contrast agents that are specifically targeted to the biochemical signatures of disease and are capable of generating sufficient image contrast[134]. Examples are presented that utilize a number of different molecular imaging quantification techniques, including measuring signal changes, calculating the area of contrast enhancement, mapping relaxation time changes or direct detection of contrast agents through multi-nuclear imaging or spectroscopy. The clinical application of CMR molecular imaging could offer far reaching benefits to patient populations, including early detection of therapeutic response, localizing ruptured atherosclerotic plaques, stratifying patients based on biochemical disease markers, tissue-specific drug delivery, confirmation and quantification of end-organ drug uptake, and non-invasive monitoring of disease recurrence. Eventually, such agents may play a leading role in reducing the human burden of cardiovascular disease, by providing early diagnosis, non-invasive monitoring and effective therapy with reduced side effects.

### Cardiovascular magnetic resonance and PET-CT of left atrial paraganglioma

Tomasian et al report a beautifully illustrated case of left atrial paraganglioma with striking PET-CT and CMR images[135].

## List of abbreviations

AR: aortic regurgitation; ASD: atrial septal defect; CMR: Cardiovascular magnetic resonance; DMD: Duchenne muscular dystrophy; EAT: epicardial adipose tissue; EF: ejection fraction; EDV: end diastolic volume; ESV: end systolic volume; HCM: hypertrophic cardiomyopathy; JCMR: Journal of cardiovascular magnetic resonance; LGE: Late gadolinium enhancement; LV: Left ventricle; MAR: myocardium at risk; MR: mitral regurgitation; MVO2: myocardial oxygen consumption rate; OEF: oxygen extraction fraction; PC: phase contrast; RV: right ventricle; TM: thalassemia major.

## Competing interests

The authors declare that they have no competing interests.

## Authors' contributions

All authors contributed to the writing of this review article.
